# Supplementary material for: Efficacy of Surgery for the Treatment of Gastric Cancer Liver Metastases: A Systematic Review of the Literature and Meta-Analysis of Prognostic Factors
Source: J Clin Med. 2021 Mar 9;10(5):1141. doi: 10.3390/jcm10051141 (PMC7963158; doi:10.3390/jcm10051141)

Study or Subgroup	log[Hazard Ratio]	SE	Weight	Hazard Ratio IV, Random, 95% CI
Baek 2013	2.31	2.99	0.2%	10.07 [0.03, 3534.38]
Garancini 2012	0.71	0.57	6.3%	2.03 [0.67, 6.22]
Kawahara 2020	−0.56	0.63	5.1%	0.57 [0.17, 1.96]
Kinoshita 2015	0.24	0.16	79.6%	1.27 [0.93, 1.74]
Nonaka 2019	−0.06	0.77	3.4%	0.94 [0.21, 4.26]
Tatsubayashi 2016	−0.17	0.77	3.4%	0.84 [0.19, 3.82]
Tsujimoto 2010	0.04	1.04	1.9%	1.04 [0.14, 7.99]
**Total (95% CI)**			**100.0%**	**1.23 [0.93, 1.62]**

Heterogeneity: $Tau^2 = 0.00$; $Chi^2 = 3.18$, df = 6 (P = 0.79); $I^2 = 0\%$
Test for overall effect: Z = 1.44 (P = 0.15)

Hazard Ratio
IV, Random, 95% CI

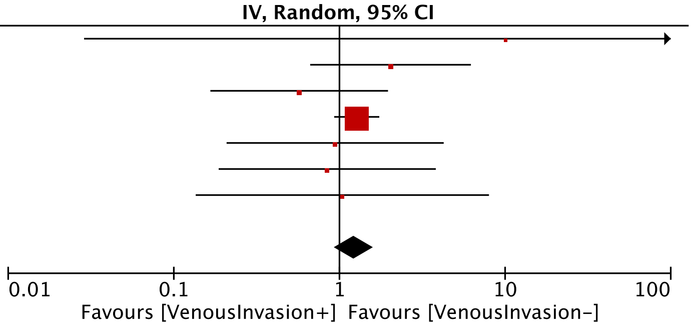

Supplement: Supplementary file 1 [file jcm-10-01141-s001.zip › jcm-1075044 supplementary/Figure S12 Forest plot of venous invasion related to OS.pdf]
