# Supplementary figures and images for: Efficacy of Surgery for the Treatment of Gastric Cancer Liver Metastases: A Systematic Review of the Literature and Meta-Analysis of Prognostic Factors
Source: J Clin Med. 2021 Mar 9;10(5):1141. doi: 10.3390/jcm10051141 (PMC7963158; doi:10.3390/jcm10051141)

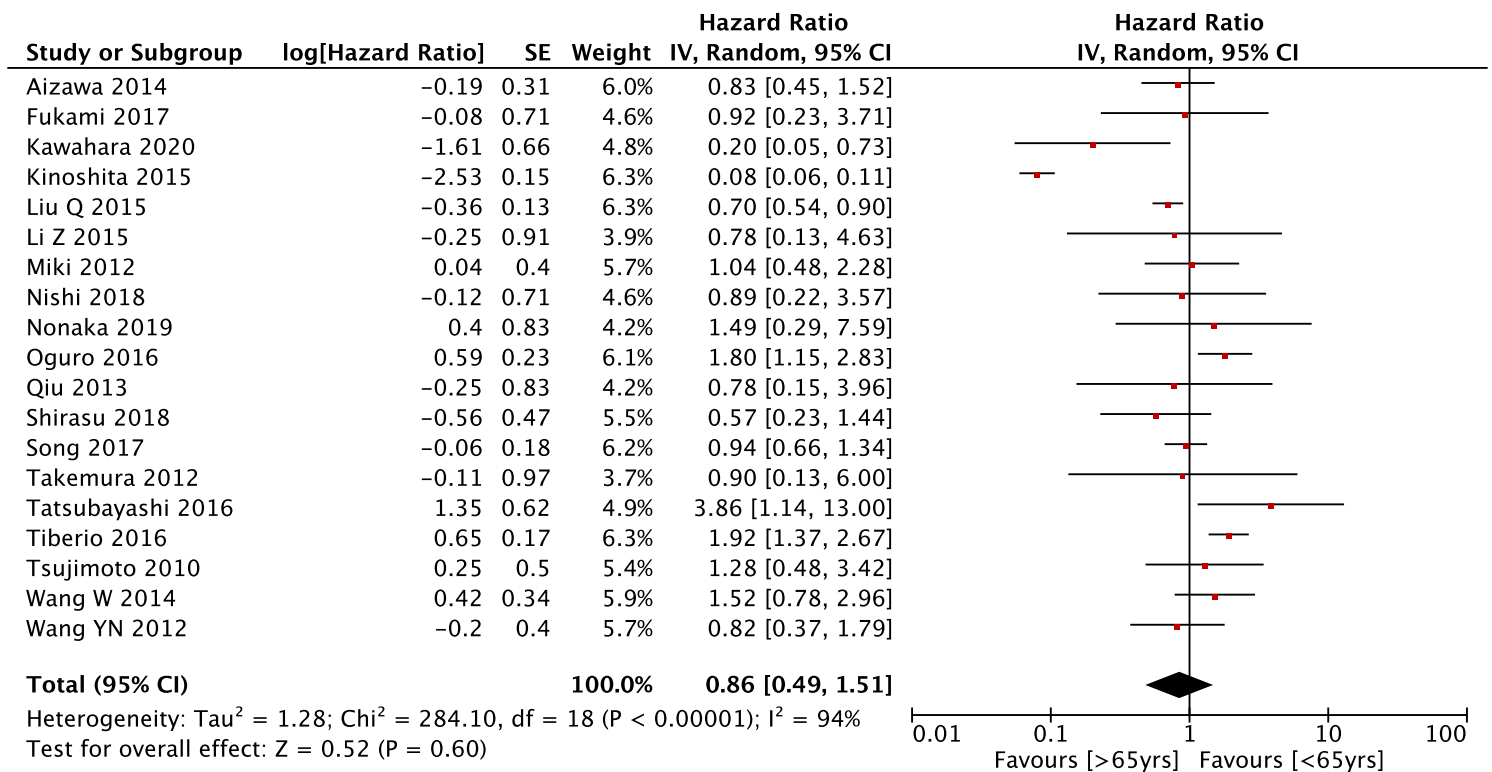

Supplement: Supplementary file 1 [file jcm-10-01141-s001.zip › jcm-1075044 supplementary/Figure S1 Forest plot of age related to OS.pdf]

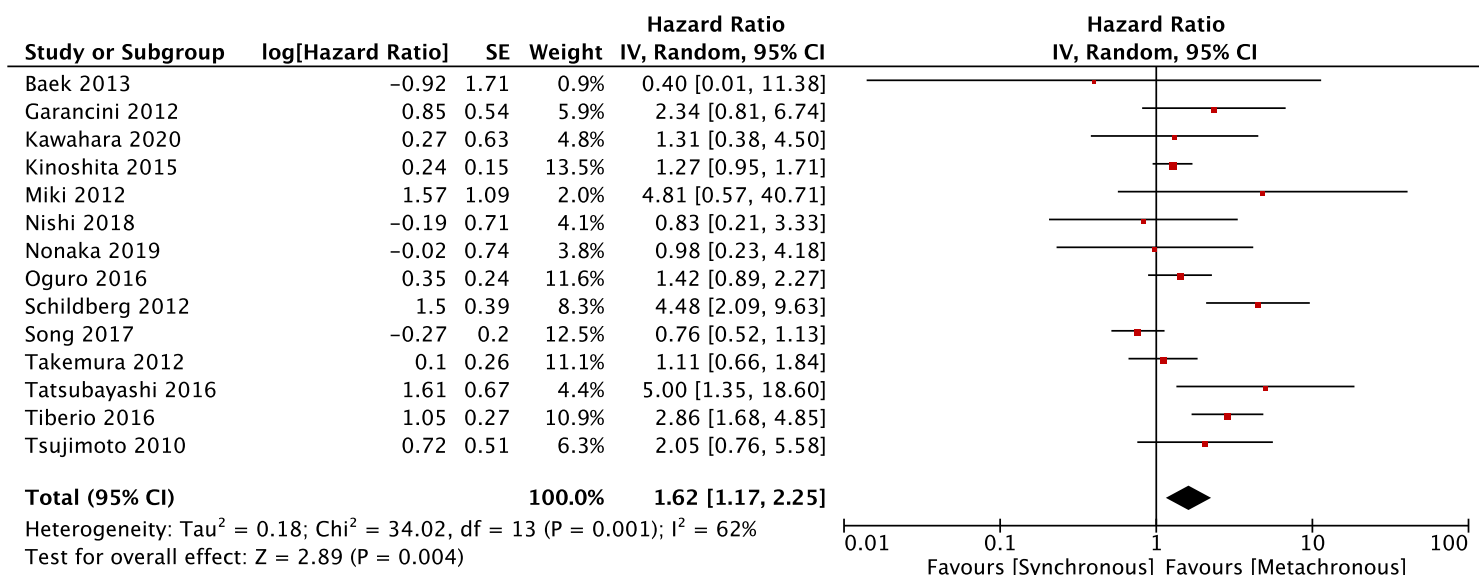

Supplement: Supplementary file 1 [file jcm-10-01141-s001.zip › jcm-1075044 supplementary/Figure S10 Forest plot of synchronous vs. metachronous related to OS.pdf]

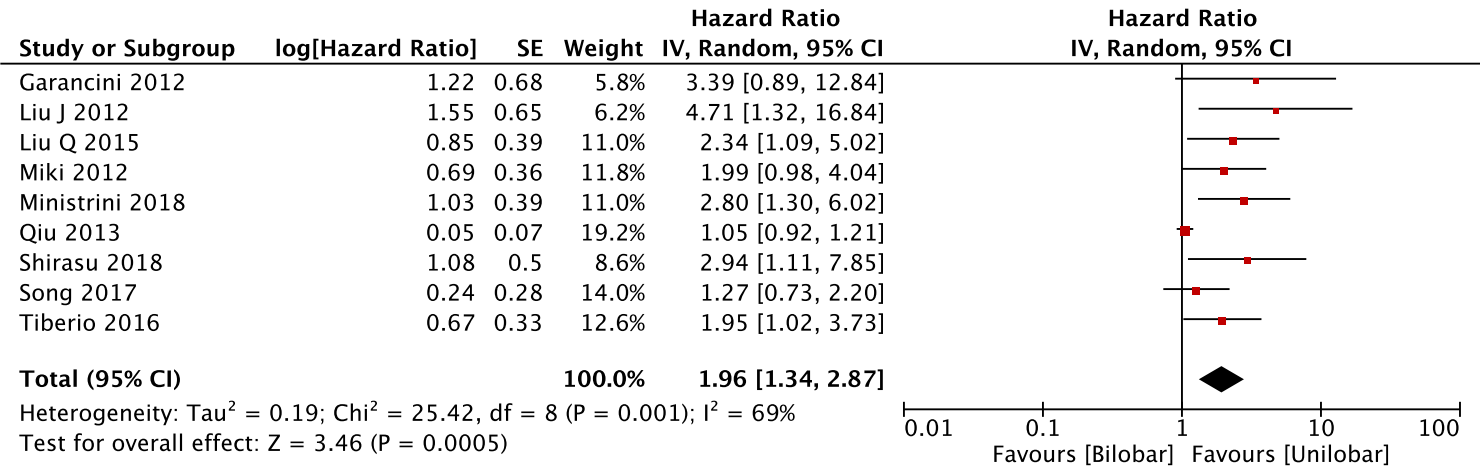

Supplement: Supplementary file 1 [file jcm-10-01141-s001.zip › jcm-1075044 supplementary/Figure S11 Forest plot of unilobar vs. bilobar related to OS.pdf]

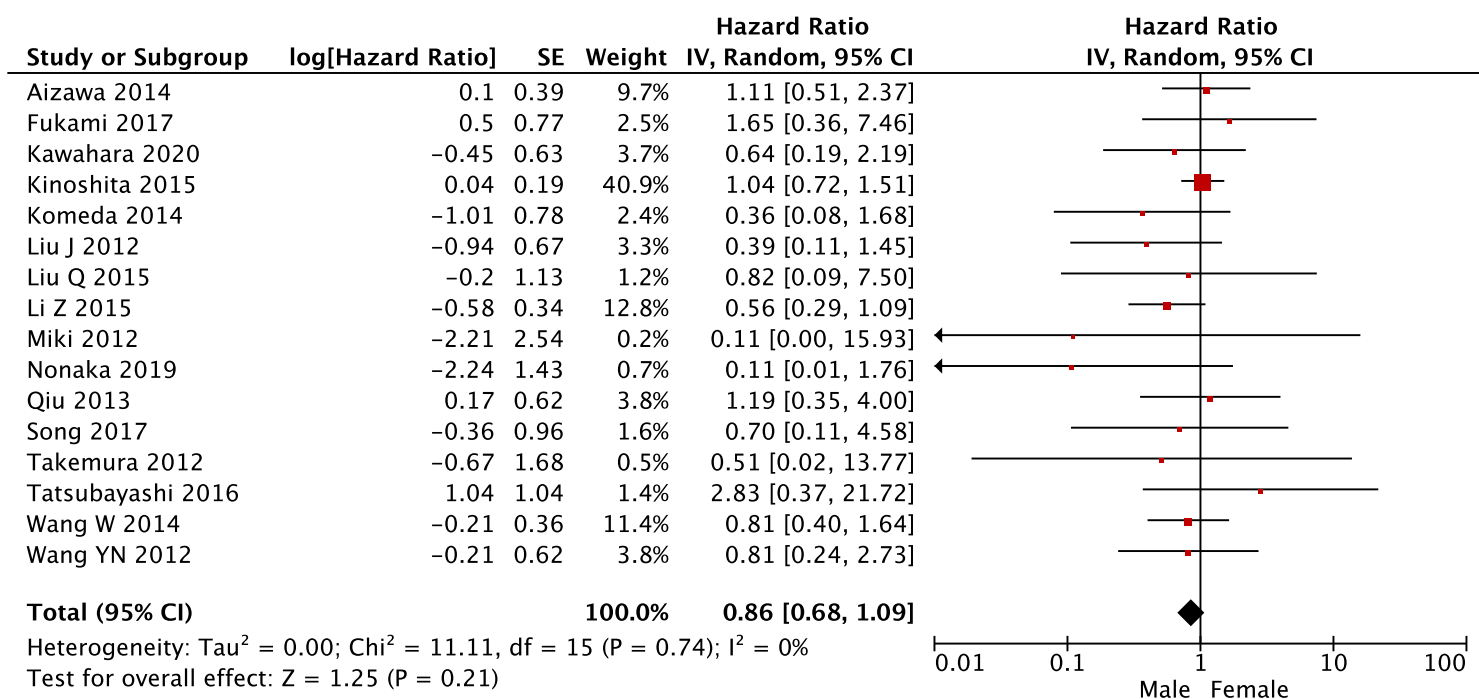

Supplement: Supplementary file 1 [file jcm-10-01141-s001.zip › jcm-1075044 supplementary/Figure S13 Forest plot of sex related to OS.pdf]

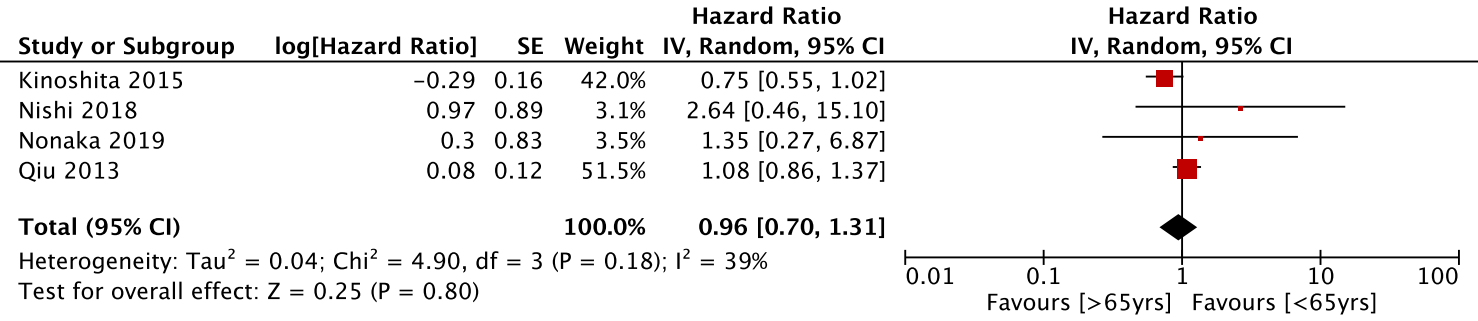

Supplement: Supplementary file 1 [file jcm-10-01141-s001.zip › jcm-1075044 supplementary/Figure S14 Forest plot of age related to DFS.pdf]

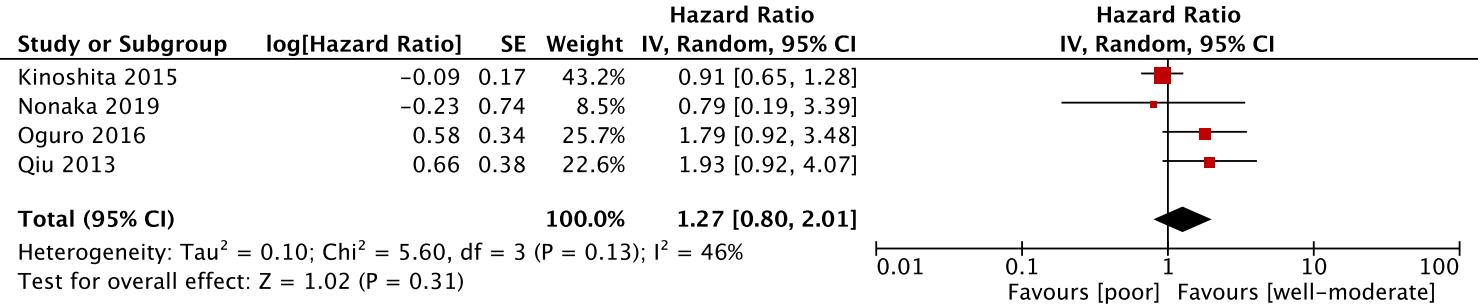

Supplement: Supplementary file 1 [file jcm-10-01141-s001.zip › jcm-1075044 supplementary/Figure S15 Forest plot of histology related to DFS.pdf]

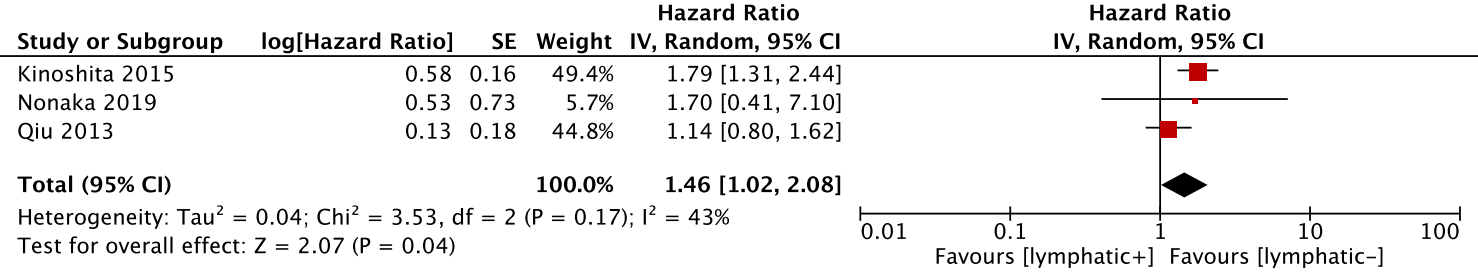

Supplement: Supplementary file 1 [file jcm-10-01141-s001.zip › jcm-1075044 supplementary/Figure S16 Forest plot of lymphatic invasion related to DFS.pdf]

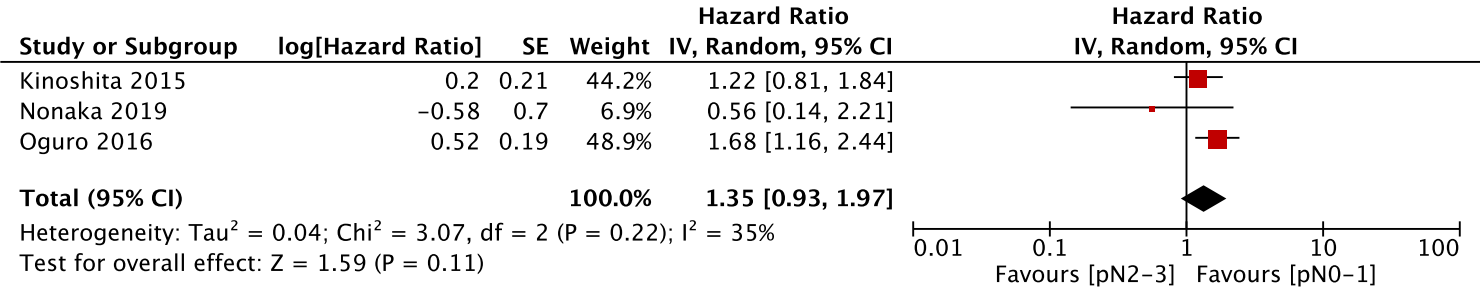

Supplement: Supplementary file 1 [file jcm-10-01141-s001.zip › jcm-1075044 supplementary/Figure S17 Forest plot of pN related to DFS.pdf]

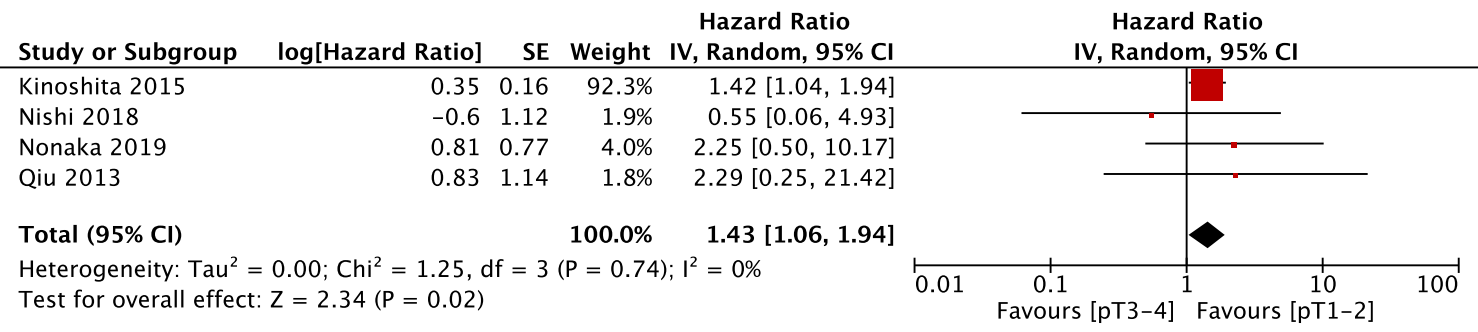

Supplement: Supplementary file 1 [file jcm-10-01141-s001.zip › jcm-1075044 supplementary/Figure S18 Forest plot of pT related to DFS.pdf]

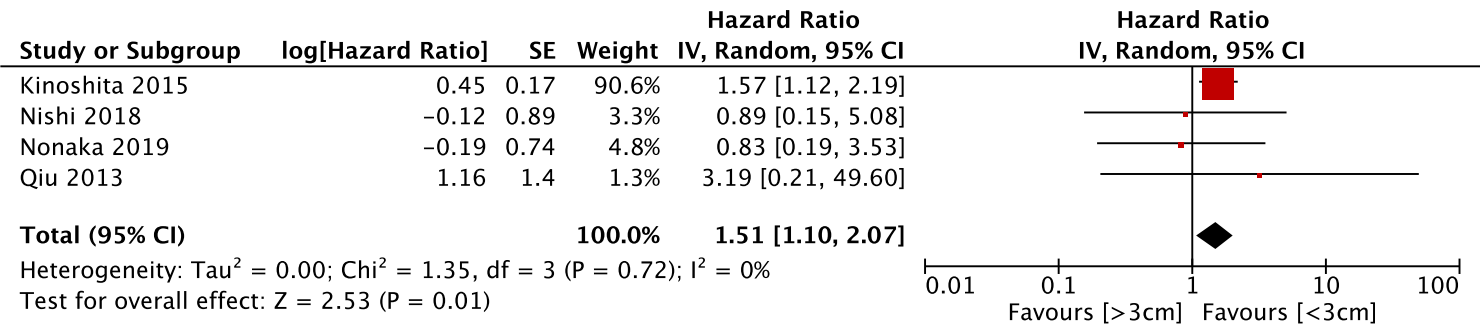

Supplement: Supplementary file 1 [file jcm-10-01141-s001.zip › jcm-1075044 supplementary/Figure S19 Forest plot of size liver mets related to DFS.pdf]

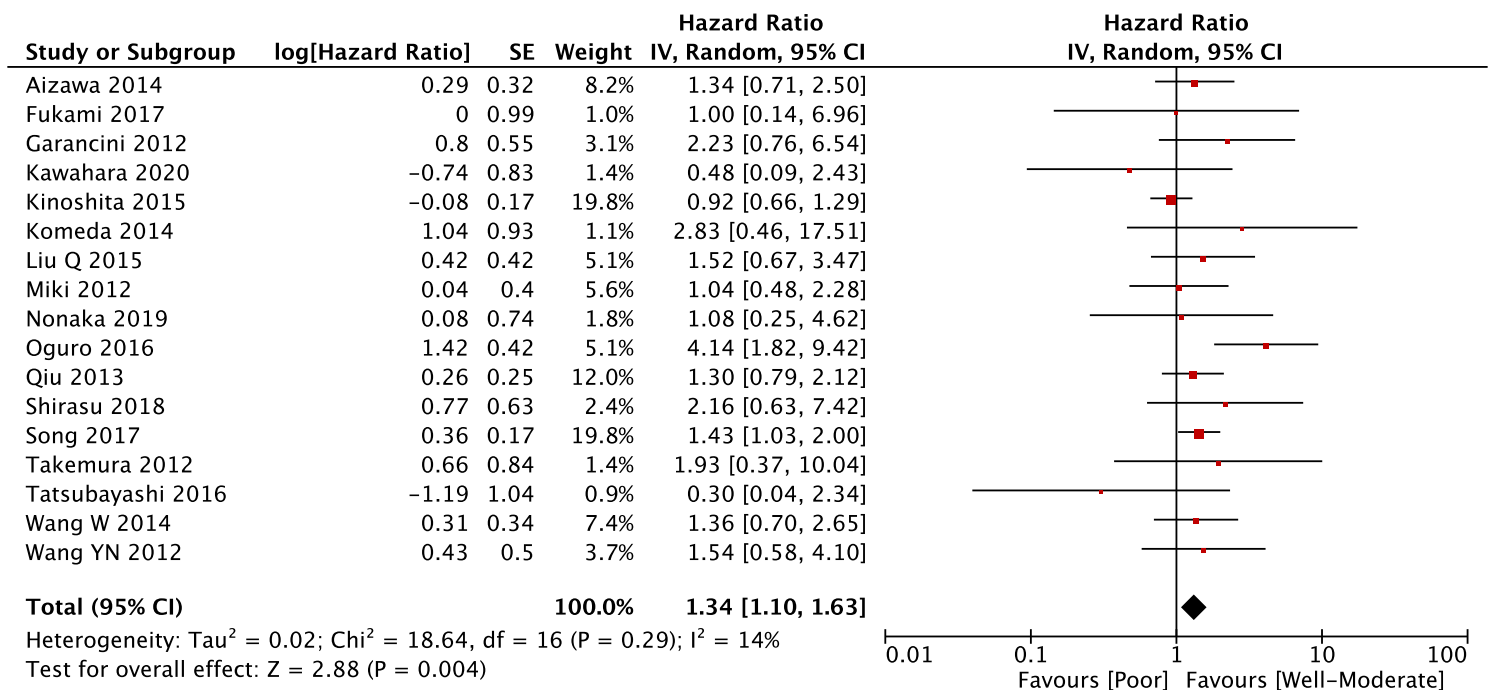

Supplement: Supplementary file 1 [file jcm-10-01141-s001.zip › jcm-1075044 supplementary/Figure S2 Forest of plot histology related to OS.pdf]

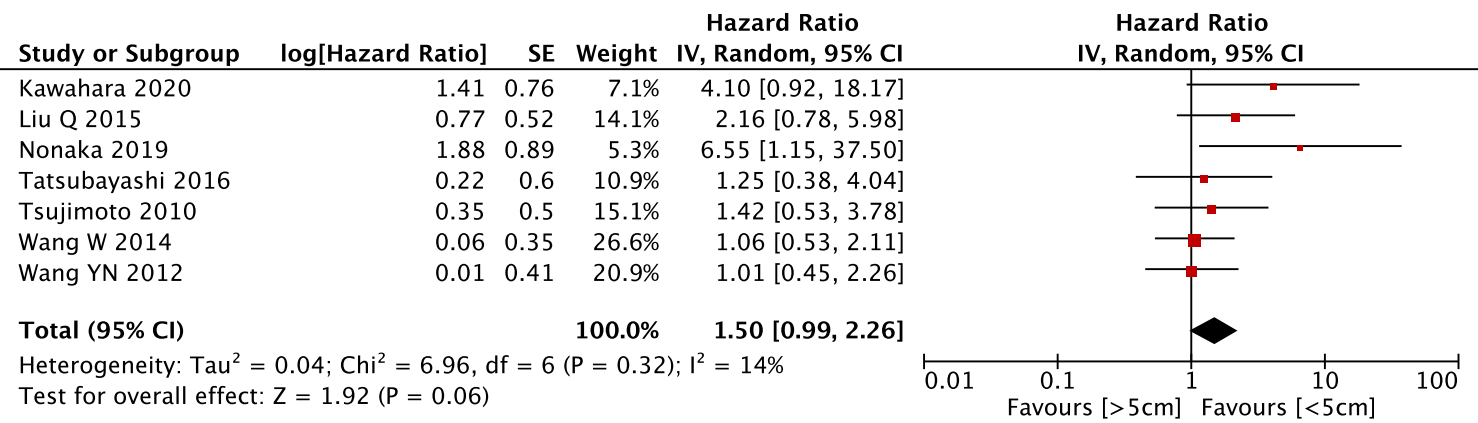

Supplement: Supplementary file 1 [file jcm-10-01141-s001.zip › jcm-1075044 supplementary/Figure S20 Forest plot of size primary tumor related to DFS.pdf]

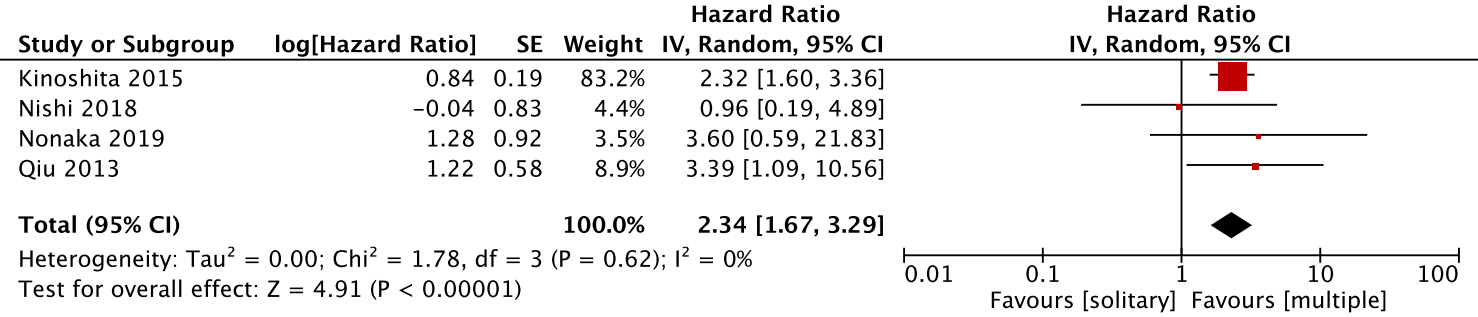

Supplement: Supplementary file 1 [file jcm-10-01141-s001.zip › jcm-1075044 supplementary/Figure S21 Forest plot of solitary vs. multiple mets related to DFS.pdf]

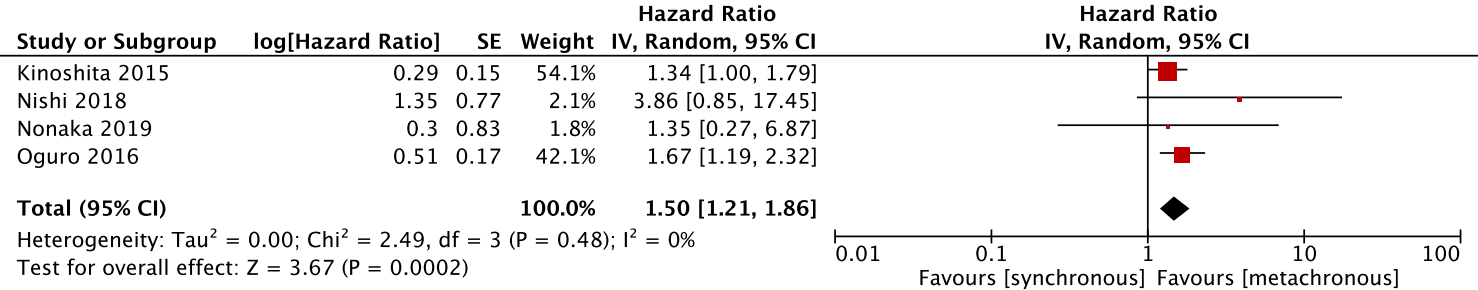

Supplement: Supplementary file 1 [file jcm-10-01141-s001.zip › jcm-1075044 supplementary/Figure S22 Forest plot of synchronous vs. metachronous related to DFS.pdf]

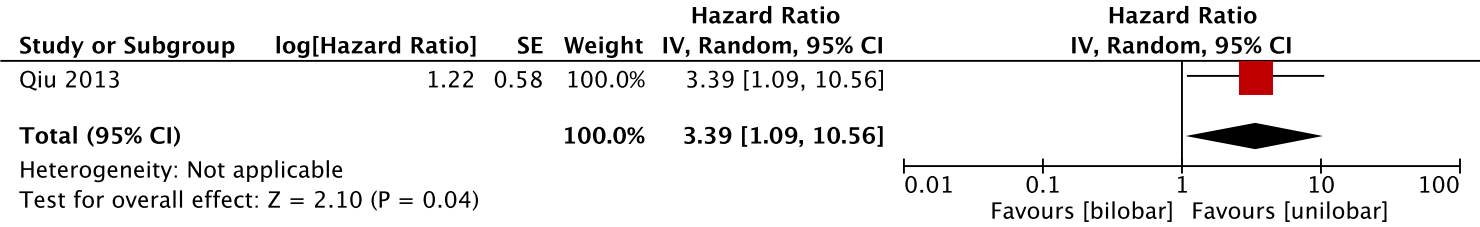

Supplement: Supplementary file 1 [file jcm-10-01141-s001.zip › jcm-1075044 supplementary/Figure S23 Forest plot of unilobar vs. bilobar related to DFS.pdf]

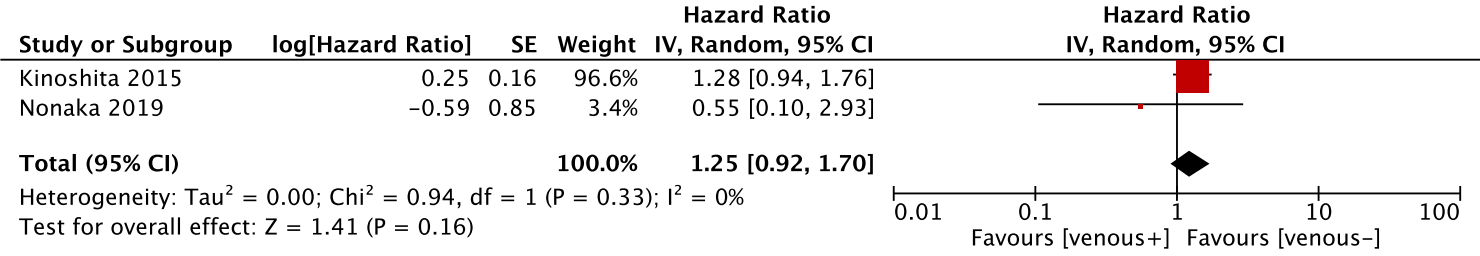

Supplement: Supplementary file 1 [file jcm-10-01141-s001.zip › jcm-1075044 supplementary/Figure S24 Forest plot of venous invasion related to DFS.pdf]

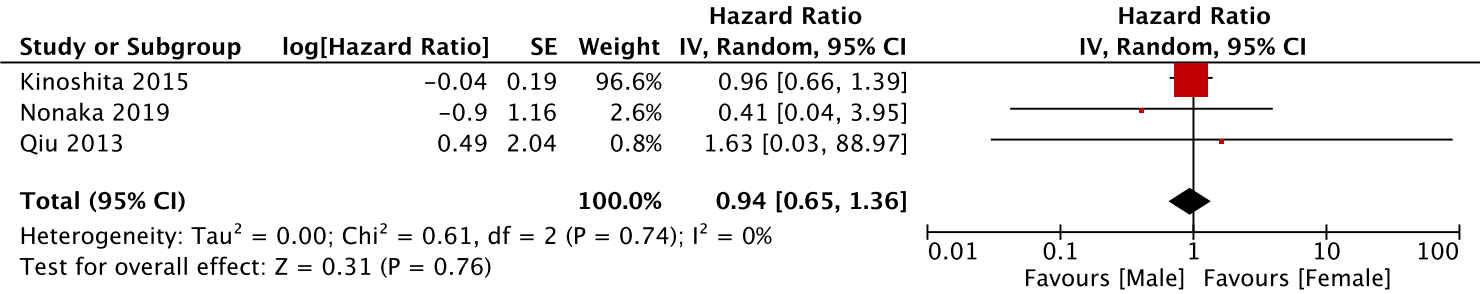

Supplement: Supplementary file 1 [file jcm-10-01141-s001.zip › jcm-1075044 supplementary/Figure S25 Forest plot of sex related to DFS.pdf]

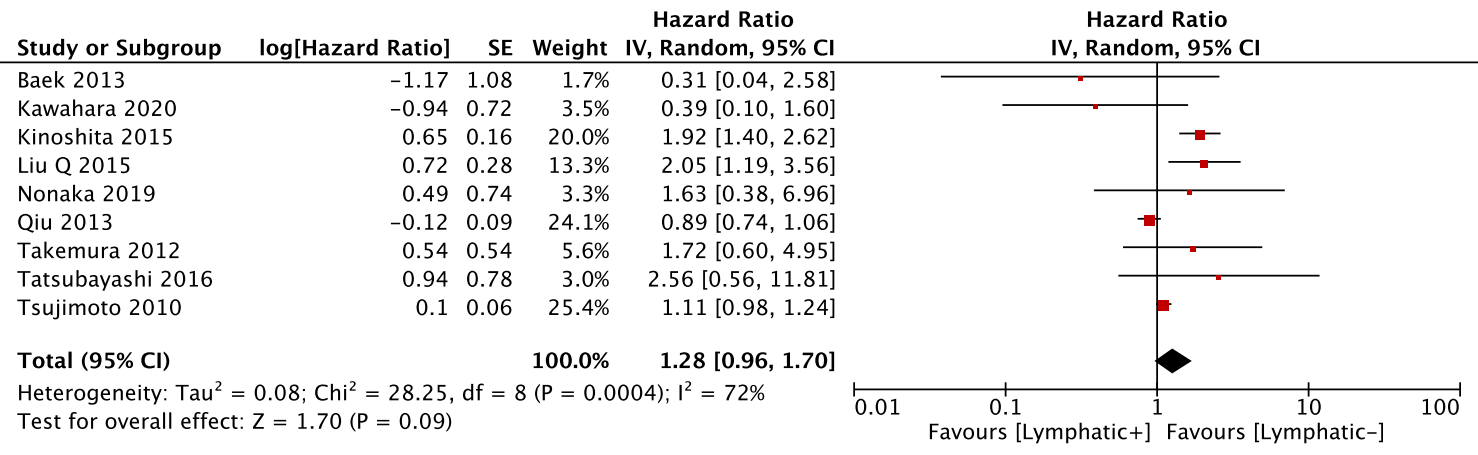

Supplement: Supplementary file 1 [file jcm-10-01141-s001.zip › jcm-1075044 supplementary/Figure S3 Forest plot of lymphatic invasion related to OS.pdf]

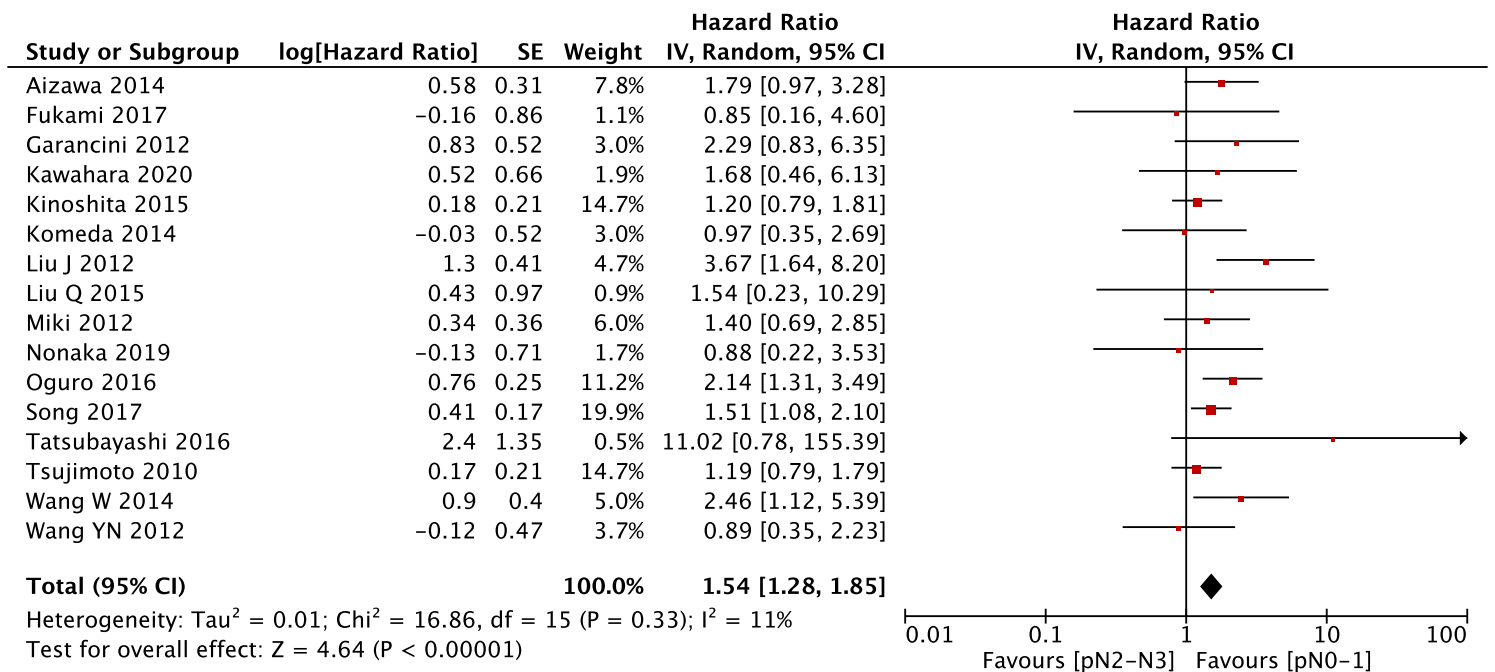

Supplement: Supplementary file 1 [file jcm-10-01141-s001.zip › jcm-1075044 supplementary/Figure S4 Forest plot of pN related to OS.pdf]

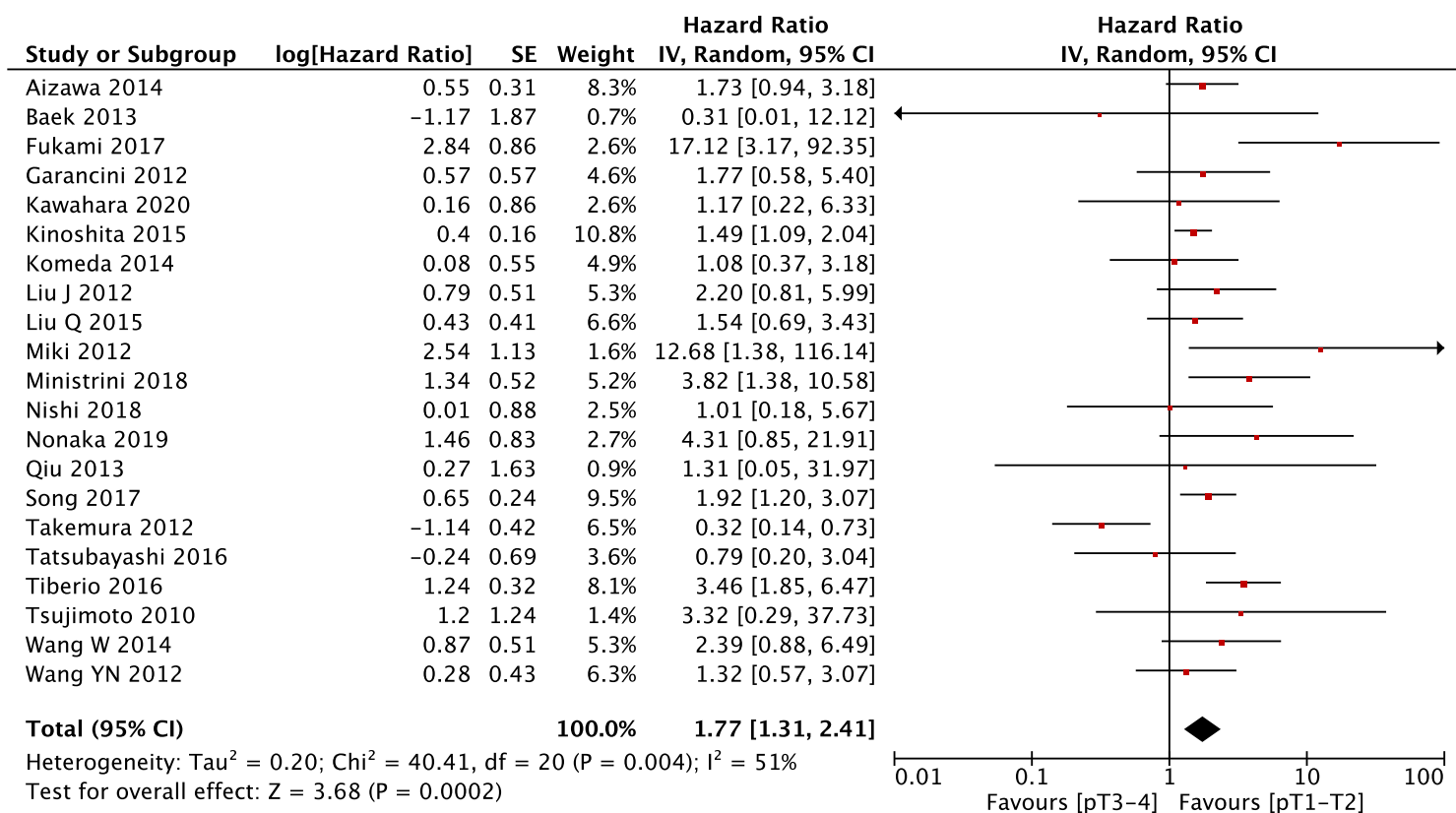

Supplement: Supplementary file 1 [file jcm-10-01141-s001.zip › jcm-1075044 supplementary/Figure S5 Forest plot of pT related to OS.pdf]

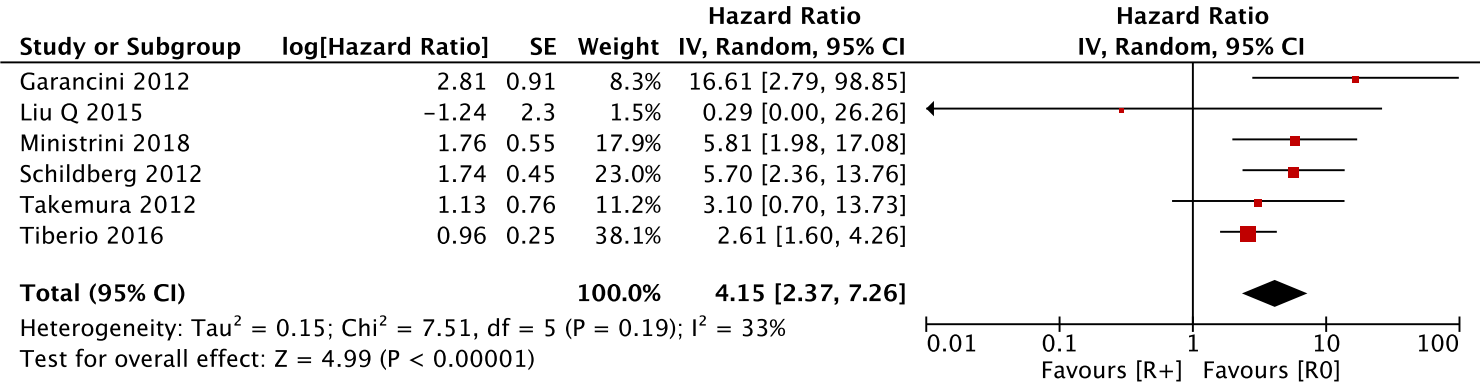

Supplement: Supplementary file 1 [file jcm-10-01141-s001.zip › jcm-1075044 supplementary/Figure S6 Forest plot of R0 related to OS.pdf]

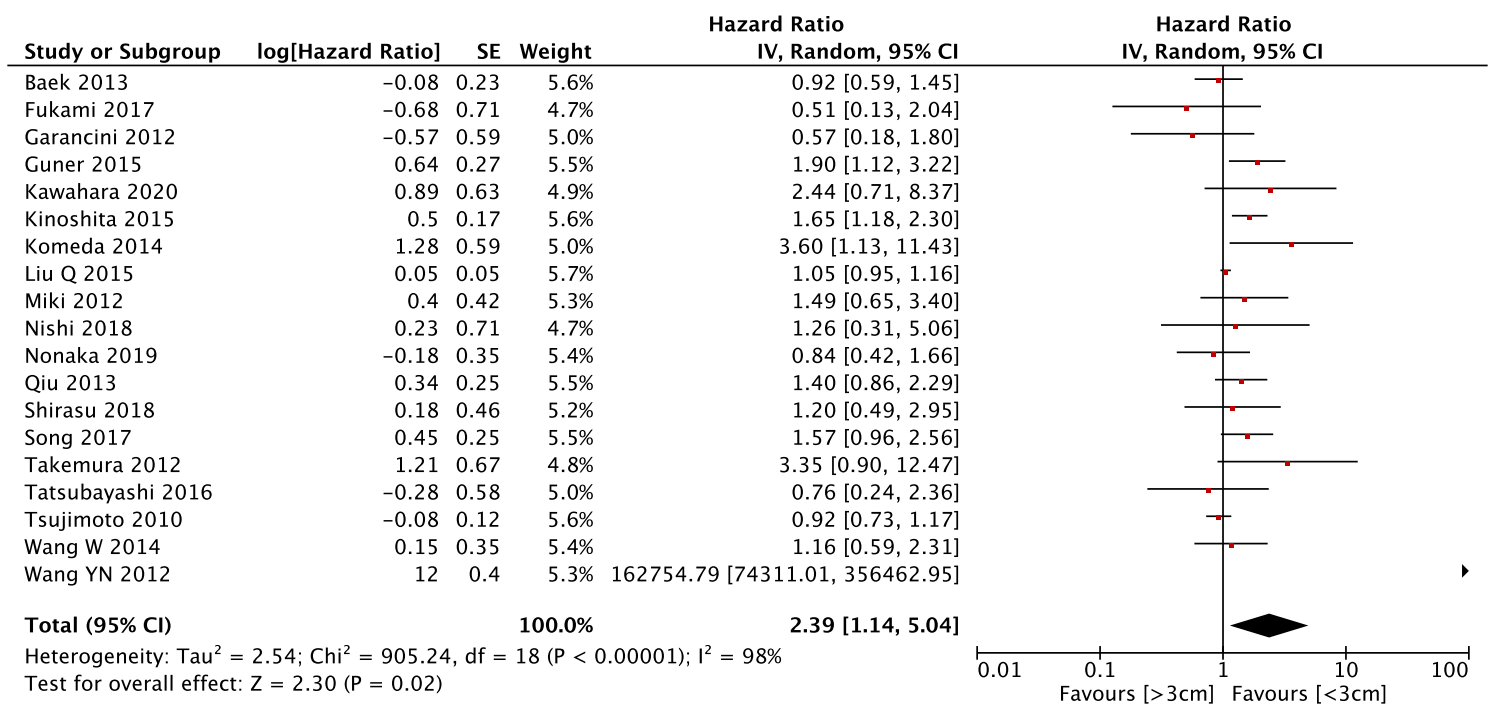

Supplement: Supplementary file 1 [file jcm-10-01141-s001.zip › jcm-1075044 supplementary/Figure S7 Forest plot of size liver mets related to OS.pdf]

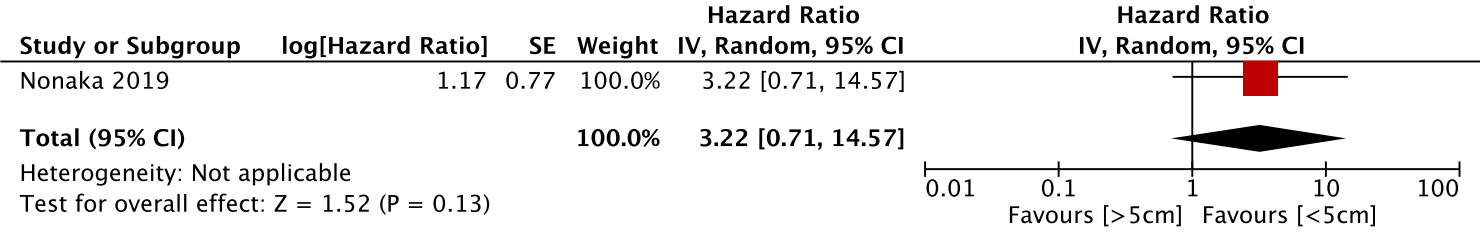

Supplement: Supplementary file 1 [file jcm-10-01141-s001.zip › jcm-1075044 supplementary/Figure S8 Forest plot of size primary tumor related to OS.pdf]

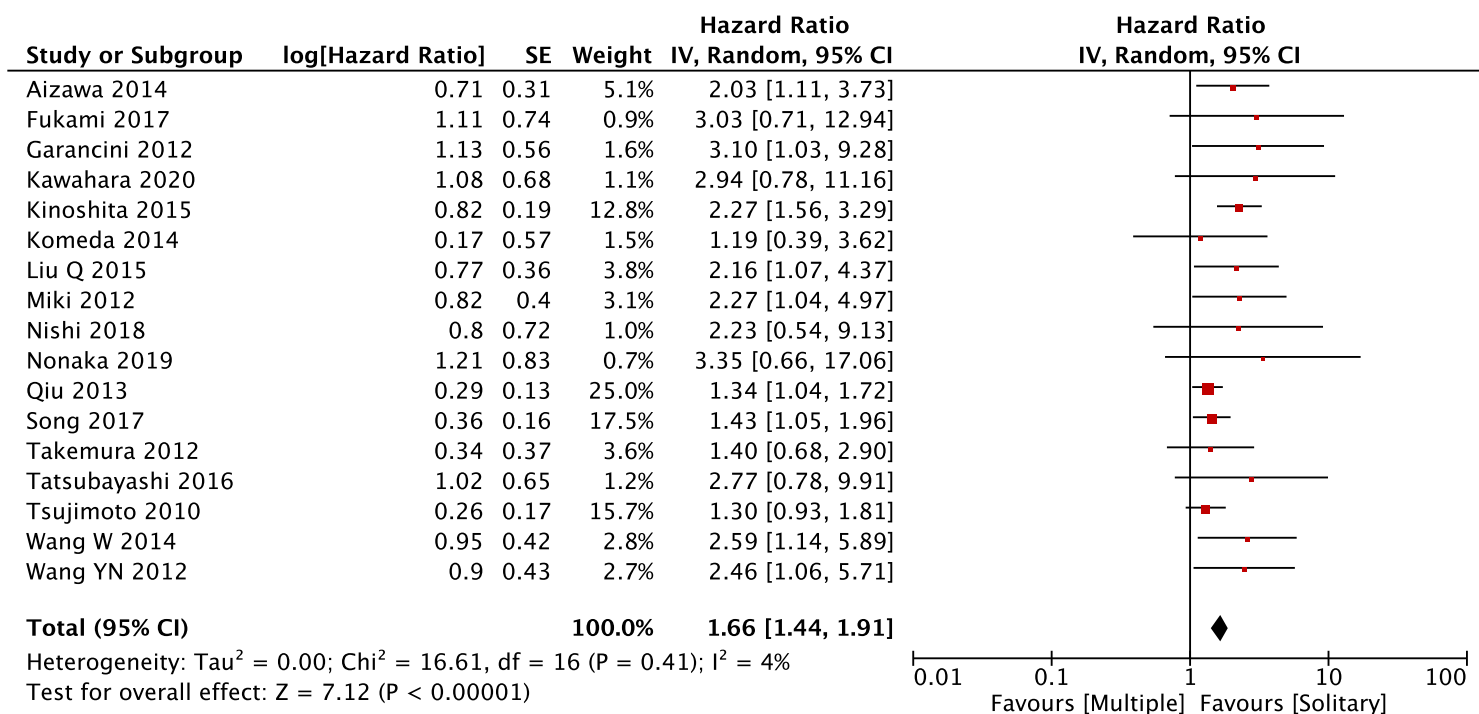

Supplement: Supplementary file 1 [file jcm-10-01141-s001.zip › jcm-1075044 supplementary/Figure S9 Forest plot of solitary vs. multiple mets related to OS.pdf]
